# Presentation of Diffuse Large B-Cell Lymphoma Relapse as a Penile Mass

**DOI:** 10.4274/tjh.2016.0132

**Published:** 2016-12-01

**Authors:** Birgül Öneç, Kürşad Öneç, Ali Ümit Esbah, Onur Esbah

**Affiliations:** 1 Düzce University Faculty of Medicine, Department of Hematology, Düzce, Turkey; 2 Düzce University Faculty of Medicine, Department of Nephrology, Düzce, Turkey; 3 Düzce University Faculty of Medicine, Department of Anesthesia and Intensive Care, Düzce, Turkey; 4 Düzce University Faculty of Medicine, Department of Medical Oncology, Düzce, Turkey

**Keywords:** Penis, Lymphoma, non-Hodgkin lymphoma, Diffuse large B-cell lymphoma, Penile mass

## To the Editor,

Penile malignant tumors constitute less than 1% of all malignancies in men but penile lymphoma is even rarer in this population [1]. Presentation with a primary penile mass is extremely rare for lymphomas, as reported only in case reports in the literature [2,3,4,5,6,7]. Here we report a case of recurrent lymphoma presenting with a penile mass lesion.

**Figure 1:**
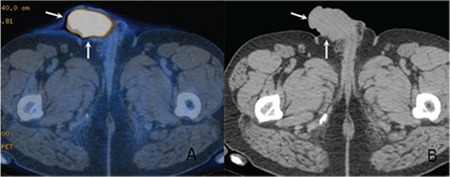
Transaxial fused positron emission tomography-computed tomography (A) and computed tomography (B) images showing the penile soft tissue mass with intense F-18 fluorodeoxyglucose uptake (arrows).

A 51-year-old man was admitted with the appearance of swelling and ulcerations of the penis that had started 2 weeks earlier. His history revealed that he was diagnosed with stage IIIB diffuse large B-cell lymphoma (DLBCL) 7 years ago, received 6 courses of R-CHOP, and was assumed to be cured after 5 uneventful years of follow-up. Swelling at the penis increased within 2 weeks with the addition of continuous pain, superficial ulcerations, and frequent and painful urination. Physical examination revealed a diffuse and indurated swelling at the shaft of the penis with an ulcer. An enlarged left inguinal lymph node was also palpable. Magnetic resonance imaging revealed a solid lesion of 55x37 mm in size, almost completely filling the penile corpus and significantly narrowing the penile urethra, extending to the glans penis. Tru-Cut biopsy of the penile lesion was consistent with DLBCL. He was staged as Ann Arbor IIIE with positron emission tomography-computed tomography revealing F-18 fluorodeoxyglucose involvement in the deep cervical left inguinal lymph nodes and a solid mass in the corpus penis (Figure 1). Treatment with R-CHOP started immediately and his complaints rapidly reduced after the first course. The patient is still having chemotherapy without complications and autologous stem cell transplantation will be considered for consolidation after complete remission.

Although most DLBCL patients have nodal presentation at admission, extranodal involvements are also common. The classical extranodal involvements sites are the breast, central nervous system, and testes. Penile involvement is a rare entity reported in case reports [2,5,7,8,9,10]. Chu et al. reviewed penile lymphomas and reported only 48 cases, among which DLBCL was the most frequent subtype with 14 cases [5]. The most common symptom of penile lymphoma was a painless mass lesion or nodule in the penis followed by ulcerations [5,7].

Surgery remains the best approach for penile cancers, whereas no standard treatment modality has been established for penile lymphomas. Systemic chemotherapy according to the subtype is a good treatment option because it preserves penile functions [2]. In our patient, R-CHOP therapy was initiated within 2 weeks after admission and obstructive symptoms were relieved immediately after the first course. Disease-free survival was reported to be between 6 and 48 months in previous case series [5], clearly indicating better outcomes than in cases of metastatic carcinomas.

In conclusion, the possibility of lymphoma involvement should be kept in mind in patients admitting with penile mass lesions, especially in patients who have a history of aggressive lymphomas, in order to avoid aggressive surgical interventions. It is important to initiate systemic chemotherapy immediately in order to prevent complications related to urethra obstruction and to preserve erectile functions.
